# Causal Economics: A new pluralist framework for behavioral economics that advances theoretical and applied foundations

**DOI:** 10.1016/j.heliyon.2019.e01342

**Published:** 2019-04-04

**Authors:** Andrew Horton

**Affiliations:** Independent researcher, 48 Thornhill Avenue, Toronto, Ontario, M6S 4C5, Canada

**Keywords:** Economics

## Abstract

This paper introduces Causal Economics, a new pluralist framework for Behavioral Economics that allows for deep incorporation of psychological drivers at a level not possible in existing models. Its core theoretical breakthrough is the replacement of conventional single-value (net cost OR net benefit), single-period exogenous lottery outcomes as utilized within mainstream economics, with endogenous multi-period outcomes that always contain both personal total benefit (B) and personal total cost (C), including certain (deliberate) and uncertain components, with cause and effect running in at least one direction. Agents optimize an overall cumulative rank dependent weighted outcome value function against internal personal psychological trade-off constraints. Its core applied breakthrough is the introduction of the Causal Coefficient and four Causal Coupling Mechanisms to evaluate and guide development of effective economic and social activities, policies and institutions. Sustainable Pareto Optimal outcomes are predicted whenever causal coupling of B and C across involved or impacted agents is achieved via a Causal Coefficient ≥ 1. It provides a powerful pluralist framework for additional research into the optimality of concepts that prioritize individual freedom of choice and responsibility to society.

## 1 Introduction

This paper introduces *Causal Economics*, a new framework for behavioral economics that significantly improves our understanding of individual decision making and its application at the macroeconomic and societal level. The model allows for deep incorporation of psychological drivers at a level not possible in existing models. It provides a powerful pluralist foundation for additional research into the optimality of concepts that prioritize individual freedom of choice and responsibility to society.

The core theoretical breakthrough of Causal Economics is the concept of *Causal Coupling*. Causal Coupling replaces conventional single-value (net cost OR benefit), single-period exogenous lottery outcomes as utilized in mainstream economics with endogenous multi-period outcomes that *always* contain both personal total benefit (B), AND personal total cost (C), including certain (deliberate) and uncertain components, with cause and effect running in at least one direction. Existing frameworks, including Behavioral Economics, cannot fully incorporate the richness of psychological trade-offs without this explicit inclusion of casual C and B in all outcomes across multi-period horizons. The core applied breakthrough of Causal Economics is the *Causal Coefficient (*X), which measures the correlation of B and C that are linked by at least one way causation across multiple periods. The model predicts sustainable Pareto Optimal outcomes whenever causal coupling of B and C across agents is achieved, reflected whenever the Causal Coefficient equals or exceeds unity.

Causal Economics builds on the insights of Cumulative Prospect Theory (Tversky and Kahneman, 1992) and Bounded Rationality (Simon, 1955) from a pluralist perspective. Agents optimize an overall weighted outcome cumulative rank dependent value function that separately weights certain (deliberate) and uncertain costs and benefits, against internal psychological trade-off constraints. The non-linear, cumulative rank-dependent value function individually weights the magnitudes of B and C by the corresponding probability weighting of each, and also cumulatively weights each event by its rank dependent overall weighted value. In Causal Economics the weighted value of the overall outcome is cumulatively transformed, rather than the probability scale as is the case in Cumulative Prospect Theory. The internal psychological trade-off constraint determines how much C an agent is willing to take on (including certain costs and risk of potential costs) in pursuit of an additional, marginal unit of B.

In mainstream economic theory, economizing is based solely on the concept of scarcity. Agents pursue their preferences by choosing from exogenous states-of-the-world that reflect allocations of scarce resources (Arrow, 1966) and maximizing expected utility. Within this context, economics becomes an allocation problem across states of nature that are imposed through equilibrium conditions (Kirman, 1989). Deviations from equilibrium are temporary distortions, not fundamental driving dynamics that explain behaviour. Causal Economics replaces the primary focus of economizing in the face of scarcity to one of bearing personal total cost to obtain resulting personal total benefit, each of which include certain (deliberate) and uncertain psychological and financial perspectives. Scarcity remains a key element of constraint, that itself differs over short-term versus intermediate-term and long-term horizons and given how it is perceived.

There are many examples of real-world decisions that cannot be effectively modelled with traditional expected utility theory nor behavioral economics in its current form. As this paper will demonstrate, such scenarios usually involve relatively certain (deliberate) initial costs over multiple periods in anticipation of subsequent benefits that are relatively larger and more uncertain. These scenarios are effectively modelled within the framework of Causal Economics. This paper will present a structured model and delineate necessary axioms, illustrating deviations from Cumulative Prospect Theory and Expected Utility Theory and addressing necessary differences in important areas such as independence, comonotonicity and decision time-horizon.

*Social Coordination Failure*, a generalization of market failure, is defined whenever there is a significant *Causal Decoupling* of C from B across agents in society. It occurs when the Causal Coefficient <1, as disincentives override incentives. The result is non-Pareto optimal allocations, where some agents bear a disproportionate amount of C and others obtain a disproportionate amount of B. Causal Economics provides a rich and fresh new theoretical lens and practical toolkit for re-examining the full range of classic and contemporary economic and social issues.

## 2 Theory

### 2.1 The need for a new theory

Many of the decisions that individuals face in applied situations do not reflect the axiomatic textbook conditions required for Expected Utility Theory to hold. The inadequacy of Expected Utility as an applied model of individual choice has been well documented (Tversky, 1975). Exhaustive reviews of experimental research have systematically demonstrated violations in each of the axioms of Expected Utility (Zafar et al., 2009).

In similar fashion, real-world decisions generally fail to align to the highly controlled conditions of the applied psychology lab experiments employed by Cumulative Prospect Theory, the dominant model of Behavioral Economics. Lab experiments have shown support for traditional rank dependence via Rank Dependent Utility (Harrison and Swarthout, 2016), but additional direct tests of Cumulative Prospect Theory have failed to show support for its rank dependence structure and cornerstone assumption of a reference frame (Bernheim and Sprenger, 2016).

It is not just for these reasons however that neither of these theories are able to fully model the most common type of significant decisions that agents face in real-world situations. Where decisions involve highly predicable (often deliberate) costs over extended periods of time, in anticipation of producing larger but less predictable benefits down the road, Expected Utility Theory and Cumulative Prospect Theory face limits. That is because they are generally based on single period, single-value lottery outcomes where agents face a distribution of outcomes that result in either a gain or a loss. Research has shown that gains and losses are not equivalent, and as a result, they can't be netted out. Specifically, losses are in general weighted twice as much as gains (Kahneman and Tversky, 1979). In line with this finding, Cumulative Prospect Theory applies separate weighting functions to benefits and costs. Causal Economics goes further to apply separate weightings to certain (deliberate) and uncertain C and B. Traditional Expected Utility and Cumulative Prospect Theory are largely unable to model real-world decisions because they don't require cost and benefit (certain or uncertain) and multiple time periods.

An applied example that highlights the problem with these models is an individual deciding to lose a significant amount of weight. This is much more than a decision about going to the gym one day and leaving with a net loss of a few pounds on that day. It requires a deliberate and sustained commitment to difficult personal costs in the form of dietary sacrifices and physical exertion that extends over months. The Causal Economics framework addresses this gap, because it includes this multi-period structure, certain (deliberate) and uncertain C and B, and requires costs to be matched to benefits. This means that costs are explicitly tied to benefits over the entire decision time-horizon. Costs are larger in earlier periods with relatively higher certainty and possible benefits are reflected in later periods with less certainty. In our example, the costs involved are things such as workouts and dietary restrictions and the gains involved are weight loss, health and self-esteem.

To further illustrate, an agent may consider exercise in order to obtain desired B (improved health, attractiveness, energy etc.) typically knowing that they can exercise more and eat better in order to achieve their goal with complete certainty if they commit. There is uncertainty surrounding the quantities of each parameter, but the agent's ability to deliberately decide and control the outcomes of their decisions is a missing element of mainstream decision-making theory. Popular culture has many expressions that capture this observed reality, such as ‘getting out what you put in’, ‘no pain, no gain’, ‘bearing the fruit of one's labour’. Research in neuroscience has also established the integration of reward and pain during choice (Talmi et al., 2009).

Table 1 illustrates how this type of decision, such as “Should I lose 25 pounds?”, compares when analyzed with Expected Utility, Cumulative Prospect Theory and Causal Economics. This type of decision spans multiple periods, includes 100% certain deliberate psychological and physical costs in early time periods (physical exertion at the gym and dietary restraint) and less certain subsequent results (the speed of actual weight loss in each period of the decision time frame). Table 1 demonstrates how Causal Economics can effectively model this situation and how Expected Utility and Cumulative Prospect Theory can't properly frame the situation, let alone explain it.

**Table 1:** An applied comparison of expected utility theory, cumulative prospect theory and causal economics.

Theory	Modelling of decision to lose 25 pounds
Expected utility theory	*Result is Inconclusive.* Decision will reflect the expected value of the range of net values (negative and positive) for a single period. Costs and benefits don't get weighted differently and all inputs are uncertain. There are no deliberate/certain inputs. There is no longer-term goal decision, just daily one-offs based on the dominant value in the set of factors above. Decisions are still a ‘lottery’ with no ‘ownership’ of the costs required to produce results.
Cumulative prospect theory	*Result is Inconclusive.* Decision will reflect the expected value of the range of net values (negative and positive) for a single period, taking into account rank dependence and a relative reference point. Net cost and benefit will be weighted differently, but all inputs are considered uncertain. There is no longer-term goal decision, just daily one-offs based on the dominant value in the set of factors above. Decisions are still a ‘lottery’ with no ‘ownership’ of the costs required to produce results.
Causal Economics	*Result is Conclusive.* Decision will weight certain and uncertain costs and benefits differently, over the entire relevant time frame. There is a longer-term goal decision, followed by daily one-offs. Decisions are not a ‘lottery’. There is the need for deliberate ‘ownership’ of the costs required to produce results.

The following assumptions apply to Table 1:

• *Positive Uncertain Value* = possible weight loss from workout (for all agents)
• *Positive Uncertain Value* = psychological satisfaction of invigorating workouts (for agents that love fitness)
• *Positive Uncertain Value* = psychological value of healthy eating (for agents that love healthy diets)
• *Negative Certain Value* = psychological cost of restrictive diet (for agents that don't love healthy eating)
• *Negative Certain Value* = psychological cost of physical exertion (for agents that don't enjoy fitness)

Real-world decision-making requires agents to weigh opportunity versus risk in an uncertain world where upfront effort is required to generate potential future benefits. Causal Economics provides a new framework that allows effective modeling of the complex decisions agents face in the real-world.

### 2.2 Causal Economics foundations

The context of decision making considered here is that of selecting a particular multi-period course of thought and all associated steps to act upon it. The preference modeling of Causal Economics is built in similar fashion to that presented for Cumulative Prospect Theory (Fennema and Wakker, 1997). Specifically;

*i* = *an outcome where P incrementally results from Q and/or Q incrementally results from P (Q is required for P and/or visa versa)*
*t* = *time period*
*+*, *−* = *positive overall outcome and negative overall outcome respectively*
(*+*), (*−*) = *personal total benefit and personal total cost outcome values respectively*
*w* = *probability weighting function*
*v* = *value function*
*π* = *capacity function of individual events*
*Ψ* = *rank dependent cumulative weighting of overall weighted event values*
*P*_*i*_ = *perceived magnitude of positive element in outcome i*
*Q*_*i*_ = *perceived magnitude of negative element in outcome i*
*p*_*i*_ = *perceived probability of positive element in outcome i*
*q*_*i*_ = *perceived probability of negative element in outcome i*
*P*^*C*^_*i*_ = *acceptable relative magnitude of positive element in outcome i*
*Q*^*C*^_*i*_ = *acceptable relative magnitude of negative element in outcome i*
*p*^*C*^_*i*_ = *acceptable relative probability of positive element in outcome i*
*q*^*C*^_*i*_ = *acceptable relative probability of negative element in outcome i*
*Ψ*^*C*^ = *acceptable rank dependent cumulative weighting of overall weighted event values*

Consider the finite set, S, of all conceivable potential states of the world. Each state, s_*i*_, is defined over the relevant decision time frame, for all 0≤t≤M and 0≤i≤N;

(1) $$S=\left(\begin{array}{ccc} s_{00} & \ldots & s_{n0}\\ \vdots & \ddots & \vdots \\ s_{0m} & \cdots & s_{nm} \end{array}\right)$$

The set of outcomes or consequences that occur with each state of the world in each period, *t*, is denoted by Z, and each z_*it*_ within Z contains B (positive value with its own probability) and C (negative value with its own probability) components. The primary value scale relevant to a decision-making agent is often financial, but does not have to be. Agents may also weigh alternatives based on financial impact, time impact and other highly personal factors, such as consistency with personal principles (Huber, 1974). The relevant time frame is defined as the set T(0,M), such that M is the last time period with a non-zero value for z_*i*_;

(2) $$T=\left\{t_{0},t_{1,}\ldots t_{i,}\ldots t_{M}\right\}$$

Perceived magnitude of B is *P*_*i*_, and perceived magnitude of C is *Q*_*i*_. Outcomes are therefore represented as $z_{i}=\left\{P_{i},Q_{i}\right\}$. The entire set of outcomes is represented as;

(3) $$Z=\left\{z_{0},\;z_{1},\ldots z_{i},\ldots z_{N}\right\}\ldots or\ldots Z=\left\{P_{0},\;Q_{0};\ldots P_{i},Q_{i};\ldots P_{N},Q_{N}\right\}$$

Personal total benefit and personal total cost are further broken down into their certain (deliberate) and uncertain components and considered over the entire relevant time horizon. Where subscript *A* denotes the certain element and subscript *U* denotes the uncertain but identified component, the following definition applies for the set of all outcomes, over all 0<t<M and all –k<i<N;

(4) $$Z_{i}=\left\{P_{Ait},Q_{Ait},P_{Uit},Q_{Uit}\right\}$$

such that in each period, *t*, the single period outcome value is;

(5) $$\{P_{it},Q_{it}\}=\{P_{Ait},Q_{Ait},P_{Uit},Q_{Uit}\}\;and\;P⊄Q,Q⊄P,p⊄q,q⊄p.$$

Each potential course of action an agent may take in the face of uncertainty may result in any of a number of potential states of the world. Perceived probability of the uncertain component of B in each period is *p*_*Uit*_, and perceived probability of the uncertain component of C in each period is *q*_*Uit*_. The decision-making time frame will contain the set of all periods up to the last period that contains at least one non-zero value of *p*_*Uit*_ or *q*_*Uit*_.

The Causal Economics prospect function is defined as follows, building upon the approach utilized in Cumulative Prospect Theory (Tversky and Kahneman, 1992);

(6) $$\;f:S\to Z,\;that\;maps\;S\;to\;Z,\;such\;that\ldots f\left(s_{it}\right)=z_{it}$$

Each multi-period state of the world is an event, denoted as E_*i*_, containing component values, E_*it*_ in each time period, *t*. E is a subset of S. Each event contains both perceived outcome magnitudes and associated perceived probabilities of occurrence over the relevant time period, that are interpreted via the value functions, *v*, and probability weighting functions, *w*, respectively. In the general context, when there is no concern with whether or not the overall weighted value of a particular event is positive or negative, the functions, *v* and *w* will often be written without the overall value indicator superscript. Specifically, each event incorporates both $B\left\{w^{\left(+\right)}\left(p_{Uit}\right),v^{\left(+\right)}\left(P_{Ait}\right),v^{\left(+\right)}\left(P_{Uit}\right)\right\}$ and $C\left\{w^{\left(-\right)}\left(q_{Uit}\right),v^{\left(-\right)}\left(Q_{Uit}\right),v^{\left(-\right)}\left(Q_{Ait}\right)\right\}$.

In Causal Economics, a prospect is reflected through the set of potential events $\left(P_{Ait},Q_{Ait},P_{Uit},Q_{Uit},E_{it}\right)$ for all *i* and *t* relevant to the decision at hand. This differs from the representation of a prospect as $\left(z_{i},E_{i}\right)$ in Cumulative Prospect Theory (Tversky and Kahneman, 1992). In Cumulative Prospect Theory, the probability of each event, producing outcome z_*i*_, is p_*i*_. By contrast, in Causal Economics, the probability attached to each E_*it*_, is the sum of both outcome probability components, specifically $p_{Uit}+q_{Uit}$. It is not meaningful for agents to rank individual values of P_*it*_ and Q_*it*_ in order, because they cannot be separated with respect to each event that contains them. Because events can only manifest through the uncertain outcomes of prospects, the net payoff value of $P_{it}-Q_{it}$ also cannot be meaningfully ranked. It does however make sense that agents may rank in order of preference each potential event value, E_*i*_.

In Cumulative Prospect Theory, the weight-adjusted value of a particular event can be interpreted as $w\left(p_{i}\right)v\left(x_{i}\right)$ (Tversky and Kahneman, 1992). In Causal Economics, the corresponding value is;

(7) $$\begin{array}{l} E_{i}\;=\sum_{t=0}^{M}\;\left[v^{+\left(+\right)}\left(P_{Ait}\right)\;+v^{+\left(+\right)}\left(P_{Uit}\right)w^{+\left(+\right)}\left(p_{Uit}\right)-\;v^{+\left(-\right)}\left(Q_{Ait}\right)-v^{+\left(-\right)}\left(Q_{Uit}\right)w^{+\left(-\right)}\left(q_{Uit}\right)\right]\;\;for\;all\ldots E_{i}\;\geq 0\\ E_{i}\;=\;\sum_{t=0}^{M}\left[v^{-\left(+\right)}\left(P_{Ait}\right)\;+v^{-\left(+\right)}\left(P_{Uit}\right)w^{-\left(+\right)}\left(p_{Uit}\right)-\;v^{-\left(-\right)}\left(Q_{Ait}\right)-v^{-\left(-\right)}\left(Q_{Uit}\right)w^{-\left(-\right)}\left(q_{Uit}\right)\right]\;\;for\;all\ldots E_{i}<0\; \end{array}$$

Causal Economics requires that agents rank each overall event value relative to others, based on its overall weighted value, as opposed to the approach of Cumulative Prospect Theory, which ranks cumulative probabilities (Tversky and Kahneman, 1992). In building a cumulative functional for Causal Economics, each event, E_*i*_ is arranged in increasing order;

(8) $$E_{0}\;<\;E_{1}<\ldots <E_{i}<\ldots \;<\;E_{N}$$

On this foundation, the overall event value weighting function, Ψ_*i*,_ will be defined subsequently, based on the difference in the capacities (non-additive generalization of relative overall weighted value) (Choquet, 1953), of E_*i*_ and E_*i+1*_ as they both relate to ΣE. The decision-making mechanism itself is an endogenous process (Langlois, 1989), whereby agents must impose a mental model on a situation where they cannot observe underlying actual empirical outcome alternatives. Agents have to consider and consolidate a vast number of unknown potential alternative states of the world and associated probability distributions, into a group of manageable categories (Thaler, 1999), represented by $w^{\left(+\right)}\left(p_{Uit}\right),w^{\left(-\right)}\left(q_{Uit}\right),v^{\left(+\right)}\left(P_{Uit}\right),v^{\left(+\right)}\left(P_{Ait}\right),v^{\left(-\right)}\left(Q_{Uit}\right),v^{\left(-\right)}\left(Q_{Ait}\right)$ and overall weighting function Ψ_*i*_. This incorporates the demonstrated psychological principle of *compartmentalizing* (Showers, 1992); a process based on each agent's own experience and judgement, which may or may not have much correlation to any actual empirical values that result.

The variables $P_{Ait},Q_{Ait},P_{Uit},Q_{Uit},E_{it},{p_{U}}_{it},{q_{U}}_{it}$ are each subjective variables that only take on values as interpreted by the decision-making agent via the probability weighting functions, $w^{\left(+\right)}\left({p_{U}}_{it}\right),w^{\left(-\right)}\left({q_{U}}_{it}\right)$, the value functions $v^{\left(+\right)}\left(P_{Ait}\right),v^{\left(+\right)}\left(P_{Uit}\right),v^{\left(-\right)}\left(Q_{Ait}\right),v^{\left(-\right)}\left(Q_{Uit}\right)$ and Ψ_*i*_. None of these variables exist in an empirical system absent of some agent's interpretation. Agents must make a judgement about the most likely value and range of values that can occur for each. The functions *w*, *v* and *Ψ* all incorporate a monotonic time discounting element that places higher weighting on more immediate B and C. When making decisions, agents make judgements regarding those components of C and B that they feel certain about and also those they feel uncertain about. For the latter, they must form a probability weighted expectation.

Comonotonic prospects provide rank-dependence and preserve the same desirability ordering of states of nature (events) in all prospects, ensuring that preferences do not depend on common consequences, unless a common consequence, when substituting it in place of another, alters the rankings of the outcomes (Wakker and Tversky, 1993). Sign-dependence allows different decision weights to be applied to events that result in incremental gain versus those that produce incremental loss (Chateauneuf and Wakker, 1999). The sign-comonotonic trade-off specification essentially allows meaningful value comparison of prospects as long as they have the same sign and are also comonotonic (Köbberling and Wakker, 2003). This criterion is important because it ensures that the weight attached to each state is the same for all prospects (Wakker and Tversky, 1993).

In order to model the full set of rich inferences possible in Causal Economics, such as optimism, pessimism, risk appetite, diminishing sensitivity and time value discounting, and in order to ensure consistent preference inferences, since weightings differ for the various components of weighted event values, the conditions of rank dependence and sign-dependence at the weighted event value level are generalized into the concept of *sign-tiered-comonotonic trade-off consistency* (STCTC). This criterion requires direct and relative comonotonicity. Specifically, $w^{\left(+\right)}\left(p_{Uit}\right),v^{\left(+\right)}\left(P_{Ait}\right)$ and $v^{\left(+\right)}\left(P_{Uit}\right)$, are comonotonic increasing, $w^{\left(-\right)}\left(q_{Uit}\right),v^{\left(-\right)}\left(Q_{Uit}\right)$ and $v^{\left(-\right)}\left(Q_{Ait}\right)$ are comonotonic decreasing, and each of the elements that comprise event values, $w^{\left(+\right)}\left(p_{Uit}\right),w^{\left(-\right)}\left(q_{Uit}\right),v^{\left(+\right)}\left(P_{Ait}\right),v^{\left(+\right)}\left(P_{Uit}\right),v^{\left(-\right)}\left(Q_{Uit}\right),v^{\left(-\right)}\left(Q_{Ait}\right)$, maintain the same relative ordering to each other (desirability ranking) in all states.

These conditions ensure meaningful inferences, providing for a strictly increasing value function, and strictly increasing psychological trade-off constraint. Utilization of a traditional exponential time weighting over the decision relevant time frame also effectively builds in time horizon comonotonicity. The result of this psychological foundation and the framework elaborated subsequently, is a preference ordering of prospects in Causal Economics that is ordinally ranked, transitive, complete, sign-tiered-comonotonic trade-off consistent and maintains stochastic dominance.

An agent may not be able to fully ascertain all components of B and/or C in the face of various prospects in real world decision-making scenarios. Agents must pragmatically make decisions based on the most robust understanding they can form in a particular situation. It is for this reason that Causal Economics employs rank dependence at the overall weighted outcome value, because, when a more specific breakdown of prospects is not possible at the level of certain and uncertain components, agents will ‘make do’ with their judgement of B and total C at the overall event level.

A prospect is fully represented and valued, over all relevant values of *i* and *t*, as follows;

(9) $$\;f\;=\;\sum_{t=0}^{M}\left\{\sum_{i=0}^{N}\left[v^{+\left(+\right)}\left(P_{Ait}\right)+v^{+\left(+\right)}\left(P_{Uit}\right)\;w^{+\left(+\right)}\left(p_{Uit}\right)-v^{+\left(-\right)}\left(Q_{Ait}\right)-v^{+\left(-\right)}\left(Q_{Uit}\right)w^{+\left(-\right)}\left(q_{Uit}\right)\right]\;\;+\;\;\sum_{i=-k}^{-1}\left[v^{-\left(+\right)}\left(P_{Ait}\right)+v^{-\left(+\right)}\left(P_{Uit}\right)\;w^{-\left(+\right)}\left(p_{Uit}\right)-\;v^{-\left(-\right)}\left(Q_{Ait}\right)-v^{-\left(-\right)}{\left(Q_{Uit}\right)}^{-\left(-\right)}\left(q_{Uit}\right)\right]\right\}$$

The following set of axioms characterize the preference structure of Causal Economics. These axioms rely heavily upon previously delineated axiomatization for Cumulative Prospect Theory (Wakker and Tversky, 1993; Chateauneuf and Wakker, 1999; Fennema and Wakker, 1997). Consider the set of all decision-relevant prospects, G, endowed with a connected product topology, where ≿ and ≾ are binary preference relations on G. Next, define F (F∈G) and two subsets; F^+^ that contains all prospects where f≥0 and F^−^ that contains all prospects where f<0. F is the additive subset, that allows consistent inferences about preference relations. F is defined as the subset that meets the following two primary conditions;

a. a permutation of the state space, Ф(*i*) exists, such that event values are arranged in increasing order $f\left(E_{1}\right)≾f\left(E_{2}\right){≾}_{\ldots }f\left(E_{i}\right){≾}_{\ldots \;}f\left(E_{N}\right)$, and,
b. for each prospect the positive part lives on A [ *f* ^+^ lives on A, such that f(i)≾0 for all $i\in A^{C}$] and the negative part lives on the complement of A, A^C^ [ *f* ^-^ lives on A^C^, such that f(i)≿0 for all i∈A].

Condition (a) provides for rank dependency and condition (b) provides for sign-dependency. Each prospect, *f*, as defined via Eq. (9), is assigned a value V(*f*), such that prospect *f* is preferred to or indifferent to prospect *g* if and only if V(f)≥V(g). In addition to the standard axioms of completeness, continuity and transitivity (Glimcher et al., 2008), over any period 0<t<M, for all values of –k<i<N, and for any prospects f,g∈F, the following additional axioms are defined;

#### 2.2.1 Causal coupled outcomes

(10) $$\begin{array}{l} When\ldots \sum_{t=0}^{M}v^{\left(+\right)}\left(P_{Ait}\right)+v^{\left(+\right)}\left(P_{Uit}\right)>0\ldots \;it\;holds\;\text{that}\;\ldots w^{\left(+\right)}\left(p_{Uit}\right)>0,\sum_{t=0}^{M}v^{\left(-\right)}\left(Q_{Ait}\right)+v^{\left(-\right)}\left(Q_{Uit}\right)>0\;\&\;w^{\left(-\right)}\left(q_{Uit}\right)>0\ldots and\ldots \;\;\\ \;\sum_{t=0}^{M}t\;where\;\left\{v^{\left(+\right)}\left(P_{Ait}\right)+v^{\left(+\right)}\left(P_{Uit}\right)\right\}\;\;\;>\;\;\;0\;\;\;>\;\;\;\sum_{t=0}^{M}t\;where\;\left\{v^{\left(-\right)}\left(Q_{Ait}\right)+v^{\left(-\right)}\left(Q_{Uit}\right)\right\}>0 \end{array}$$

The axiom represented by Eq. (10) requires that each outcome contains both B and C (causation) and the notion that agents in general have more immediate control over and face more immediate impact on their C in terms of upfront effort than they do over the B that typically follows with some time delay and being subject to more uncertainty due to cumulative counter agent effects—the latter is defined subsequently.

#### 2.2.2 Sign tiered-comonotonic trade-off consistency

The *sign-tiered-comonotonic trade-off consistency* (STCTC) criterion requires direct and relative comonotonicity and sign dependence. Specifically, $w^{\left(+\right)}\left(p_{Uit}\right),v^{\left(+\right)}\left(P_{Ait}\right),v^{\left(+\right)}\left(P_{Uit}\right)$ are each comonotonic increasing functions, $w^{\left(-\right)}\left(q_{Uit}\right),v^{\left(-\right)}\left(Q_{Uit}\right)$ and $v^{\left(-\right)}\left(Q_{it}\right)$ are comonotonic decreasing functions, and each of these elements that comprise event values are comonotonic independent from each other, and maintain the same relative ordering (desirability ranking) in all states. These conditions allow meaningful inferences, by ensuring consistent ordering of utility intervals. They underpin a strictly increasing value function and strictly increasing psychological trade-off constraint. Utilization of a traditional exponential time weighting over the decision relevant time frame also effectively builds in time horizon comonontonicity.

#### 2.2.3 Relative certainty preference and personal total cost aversion

Wherever all inputs other than $w^{\left(-\right)}\left(q_{Uit}\right)$ are fixed and where $w^{\left(-\right)}\left(q_{Uit}\right)$ is lower in *f* than it is in *g,* then f≻g. This axiom extends the standard concept of risk aversion to become one of *Personal Total Cost Aversion*.

A strong argument that $p_{Uit,}q_{Uit}>0$ in all outcomes is the assertion that every outcome that could materialize, would by definition preclude alternative outcomes, which means some lost opportunity and some avoided cost (Cain, 1995). The fundamental economic concept of opportunity cost illustrates very effectively that decision-making itself is a non-trivial endeavour because a decision requires an alternative (opportunity) cost to be borne, whether the decision is to act or to remain in status quo. In addition, the ever-present existence of uncertainty, a reality that itself is the major driver of the need for decision-making theory, always introduces a discomfort (C) element, whether through an explicit decision to change or to try to maintain the status quo. The latter is still a decision and is subject to the uncertainty that it might not prevail despite best efforts.

### 2.3 The Causal Economics value function

Each prospect, *f*, is assigned a value V*(f)*, such that prospect *f* is preferred to or indifferent to prospect *g* if and only if V(f)≥V(g). Each prospect represents a potential alternative decision/course of action, and as such, agents will value the set of possible prospects and select the one that results in the highest V*(f).* Where λ represents the decision to be made, it can be understood as;

(11) λ=MaxV(f)

The Causal Economics value function is a strictly increasing function $V\left(f\right)=V^{+}\left(f\right)+V^{-}\left(f\right)$, defined as follows;

(12) $$V\left(f\right)=\;\int_{t=0}^{M}\left\{\int_{i=0}^{N}\left[w^{+\left(+\right)}\left(p_{Uit}\right)v^{+\left(+\right)}\left(P_{Uit}\right)+v^{+\left(+\right)}\left(P_{Ait}\right)-w^{+\left(-\right)}\left(q_{Uit}\right)v^{+\left(-\right)}\left(Q_{Uit}\right)-v^{+\left(-\right)}\left(Q_{Ait}\right)\right]\;\Psi_{i}^{+}\;+\;\;\int_{i=-k}^{-1}\left[w^{-\left(+\right)}\left(p_{Uit}\right)\;v^{-\left(+\right)}\left(P_{Uit}\right)\;v^{-\left(+\right)}\left(P_{Ait}\right)-\;w^{-\left(-\right)}\left(q_{Uit}\right)v^{-\left(-\right)}\left(Q_{Uit}\right)-v^{-\left(-\right)}\left(Q_{Ait}\right)\right]\;\Psi_{i}^{-}\;\right\}$$

The fact that outcomes are causally coupled (contain both B and C and one or two way causation) in Causal Economics, with different probability weightings and value magnitude interpretations attributed to each, means that it is not possible to rank each value of z_*i*_ directly in increasing order. It is therefore not necessary that when $i>j,z_{it}≻z_{jt}$.

Causal Economics does not require that probabilities be transformed by a cumulative subjective weighting function in the same way that they are for Rank Dependent Utility and Cumulative Prospect Theory (Quiggin, 1992). However, in order to ensure that rank dependence, monotonicity and stochastic dominance are preserved, a cumulative transformation (Fennema and Wakker, 1997) is applied to the overall weighted value of each event, E_*i*_. In Cumulative Prospect Theory, each outcome, $z_{i},\ldots .\;z_{N}$, is ranked in increasing order with individual probabilities coupled one to one to each outcome value (Tversky and Kahneman, 1992). Weighting is then represented by a non-linear transformation of probabilities, the weight for each outcome reflecting the ‘marginal’ probability, that can be interpreted such that a particular outcome value is “likely to be at least as good as” those that precede it in the ranking (Tversky and Kahneman, 1992).

In Causal Economics, the overall weighting function is represented by the overall weighted value of each event, E_i_, relative to other potential events. The weighting applied at each value, *i*, through the function Ψ, is therefore the incremental overall weighted value obtained, reflecting that the overall weighted outcome value is “at least as good as” those that precede it in the ranking of overall weighted outcome values. The Causal Economics value function (12) is typically structured in similar fashion to that of cumulative prospect theory (CPT). Specifically, events are considered as gains or losses relative to a reference point, generally the status quo, allowing for differing attitudes toward losses and gains (Tversky and Kahneman, 1992).

Risk aversion and variability seeking behaviour will be discussed subsequently in detail, but at this point two key traits of the Causal Economics value function (12) can be identified, taken from Cumulative Prospect Theory, that do underpin inherent overall risk aversion in decision-making agents, ceterus paribus. Firstly, the losses portion of the curve is convex, implying that people are motivated more by losses than by gains and as a result will devote more energy to avoiding loss than to achieving gain (Tversky and Kahneman, 1992). This also captures diminishing sensitivity toward B. Secondly, the value function is steeper for losses than it is for gains (Kahneman et al., 1991).

The following weighting functions are defined in Causal Economics, assigning a weight based on the difference between two capacity functions (Choquet, 1953);

(13) $$\begin{array}{l} \;\Psi_{i}^{+}=\pi^{+}\left(E_{i+1}\;/\sum_{i=-k}^{N}E_{i}\;+\ldots +E_{N}/\sum_{i=-k}^{N}E_{i}\right)-\pi^{+}\left(E_{i\;}/\sum_{i=-k}^{N}E_{i}+\ldots +E_{N}/\sum_{i=-k}^{N}E_{i}\right)\;\;for\;all\ldots 0\;\leq i\leq n-\;1\\ \;\Psi_{i}^{-}\;=\;\pi^{-}\left(E_{-k}\;/\sum_{i=-k}^{N}E_{i}\;+\ldots +E_{i}/\sum_{i=-k}^{N}E_{i}\right)–\pi^{-}\left(E_{-k\;}/\sum_{i=-k}^{N}E_{i}+\ldots +E_{i-1}/\sum_{i=-k}^{N}E_{i}\right)\;\;\;for\;all\ldots –k+1\leq i\leq 0\\ \;\Psi_{i}^{+}=\;\;\pi^{+}\left(E_{N}/\sum_{i=-k}^{N}E_{i}\right)\;\;\;\ldots \;\;\;for\;i=n\;\;\;\;and\;\;\;\;\Psi_{i}^{-}\;=\;\left[\;\pi^{-}\left(E_{-k}/\sum_{i=-k}^{N}E_{i}\right)\;\right]\;\;for\;\ldots \;i=-k \end{array}$$

The cumulative weighting function, Ψ, employed in Causal Economics (13), shares a number of structural characteristics with the probability weighting function utilized in CPT. Most notably it is S-shaped, concave near zero and convex near its positive and negative end points (Tversky and Kahneman, 1992). Outcomes close to the status quo get less weighting relative to extreme values. In CPT, the S-shape of the probability weighting function implies that small probabilities are generally over weighted and that moderate to high probabilities are generally under weighted (Tversky and Kahneman, 1981). As a result, in CPT, *risk aversion* behaviour is typical for gains of high probability and losses of low probability, whereas *variability seeking* behaviour is typical for gains of low probability and losses of high probability.

In Causal Economics, the s-shape of Ψ, shown in Eq. (13), implies a relative over weighting of small probabilities and higher outcome magnitudes and a relative under weighting of high probabilities and smaller outcome magnitudes. This asymmetry produces relatively high risk aversion for low probability losses with large magnitude and for high probability gains with small magnitude. Agents are generally very intimidated by large potential losses, even when the probability of occurrence is low. They will also typically not pursue risk to obtain gains that are small, even if the probability of attainment is large, as they just don't see a worthwhile ‘big payoff’.

This asymmetry produces variability seeking behaviour for large magnitude gains with low probability and for small magnitude losses of high probability. As an illustrative example, agents often prefer to forego high probability low to moderate magnitude gains in pursuit of large, but remote upside payoffs; they are willing to gamble in giving up some certain positive outcome for a chance at significantly larger upside. Agents are also often very willing to take any chance they can to avoid loss, bearing significant risk of a large negative outcome in order to avoid an even higher likelihood or completely certain smaller loss.

Because the Causal Economics weighting function includes multiplication by outcome magnitudes, it's S-shape will be ‘vertically stretched’ relative to the s-shape of the probability weighting function employed in CPT. The trade-off dynamics concerning sensitivity and variability attitudes discussed to this point do not explicitly incorporate the impact of time horizons and sustained effort on the B/C trade-off. Over longer time horizons, the psychological trade-off constraint transitions from generally concave to convex and back to concave, as sensitivity and risk aversion interplay with cumulative counter agent effects.

### 2.4 The psychological constraints

Causal Economics postulates that agents optimize by making trade-off decisions in the face of their own internal psychological constraint of acceptable B relative to causally coupled C. The addition of a psychological trade-off constraint is a significant diversion from traditional economic thought on how agents make decisions. In the approach of Causal Economics, external constraints, such as budget, are always considered to have their impact on utility indirectly, through interpretation by the agent, based on that agent's personal psychological constraint. The constraint is a matter of choice (Vahabi, 2001). Consider for example the constraint of a particular current budget that is very low relative to the purchase desires of an agent. Some agents may choose to lower consumption to compensate, some may borrow, and some may shift additional consumption to investment in an attempt to raise the budget constraint over time.

Real world decisions occur in an environment where agents face an aggregation of correlated and uncorrelated factors and must sort through them as best as they can. They make endogenous decisions in the face of their bounded memory capabilities, potentially following a randomized (Cover and Hellman, 1970) or deterministic process (Montew and Said, 2010). Agents must weigh opportunity versus risk in an uncertain world and decide on a trade-off that meets their personal comfort level, bearing *both* C and B (with one or two way causation and potentially certain and uncertain elements) in all outcomes at some point in the time frame relevant to the decision and its associated actions. Popular culture has many expressions that capture this observed reality, such as ‘getting out what you put in’, ‘no pain, no gain’, ‘bearing the fruit of one's labour’. Research in neuroscience has also established the integration of reward and pain during choice (Talmi et al., 2009).

Mainstream approaches to decision-making generally do not capture this fundamental element because they utilize single-period, single-value outcome lottery experiments, an approach that does not capture the underlying conditions of decision-making. Whereas the traditional budget constraint allows tangible trade-off through observed prices, the psychological trade-off constraint reflects an individual's ‘personal psychological trade-off terms’ or ‘price’, capturing how willing they are to bear C to obtain B. Each agent formulates these expectations based on their set of life experiences. Life experiences impact each individual agent's anticipation of magnitudes $\left[v^{\left(+\right)}\left(P_{Uit}\right),v^{\left(-\right)}\left(Q_{Uit}\right),v^{\left(+\right)}\left(P_{Ait}\right),v^{\left(-\right)}\left(Q_{it}\right)\right]$ and probability weightings $\left[w^{\left(+\right)}\left(p_{Uit}\right),w^{\left(-\right)}\left(q_{Uit}\right)\right]$ in all prospective situations as well as the acceptable overall level of B vs. C—defined as B^C^ vs. C^C^. The slope of the trade-off curve therefore represents the marginal rate of B that is required in order to take on an additional unit of C.

The nature of the optimization decision will be elaborated subsequently, but at this point it is helpful to clarify the distinct roles played by the value function, *v*, and the psychological trade-off constraint, *c*. The value function represents an agent's judgement of the particular prospects they face when making a decision, in terms of what specific events may occur and what particular B and C are associated with each event. The value function compares the particular prospects, via their component events, that the decision-maker anticipates they may face with some likelihood. By contrast, the psychological trade-off constraint directly conveys a decision-maker's underlying preferences, depicting the amount of B that is required in order for an agent to take on an additional unit of C or similarly the C that an agent will bear in order to obtain an additional unit of B. The concept of the $B^{C}/C^{C}$ ratio is therefore defined by the psychological trade-off curve, which is also dependent upon the overall outcome value E, necessitating weighting by Ψ^C^_*i*_. The resulting optimization dynamic between *v* and *c* is therefore one in which anticipated prospects are evaluated, via *v*, and compared to acceptable scenarios, via *c*.

Prospect Theory (Kahneman and Tversky, 1979) and Cumulative Prospect Theory (Tversky and Kahneman, 1992) aggressively introduced the powerful tools of psychology to economic decision-making. Causal Economics follows on that path, recognizing in addition that agents' own internal associations of C and B serve as a constraint similar to a budget constraint in traditional models. The approach of Causal Economics essentially expands the purely scarcity driven need for trade-off to a broader need for trade-off based on the full psychological drivers of the agent. It can seem counterintuitive that agents would impose a constraint on themselves; given rational expectations theory (Lucas and Sargent, 1981), it seems rational that an agent would immediately redefine their C and B associations so that they could increase B and reduce C. Such a redefinition of preference associations is in fact possible through dynamic inconsistency (Strotz, 1955), but typically takes extended periods of time, faces strong internal resistance and requires significant incentive (Kahneman et al., 1991). An agent's acceptable level of C^C^ typically includes a selection about what price to pay, what certain effort to put forth and what level of risk to bear.

In a theoretical world where agents can have whatever they want without cost they would be able to obtain infinite utility, or V(f)=∞. However, the real world presents agents with a very different set of circumstances, reflecting two primary external ‘barriers’ to the infinite utility scenario. The first is the constraint of physical world factors, including scarcity, such as the fact that food needs to be created at some point before it can be eaten. The second is the constraint that the decision-maker cannot act unilaterally; they must interact with other agents that control resources, possess their own views, and are in turn seeking to maximize their own utility. This second type of constraint is captured in the concept of *Cumulative Counter Agent Effects*. Whether a decision-maker thinks the position of potential counter parties is rational or not, the decision-maker generally knows that they can only influence it, that they must essentially take the position as a given and optimize against it as an ‘almost exogenous’ input in the short to intermediate term.

***Cumulative counter agent effects***

In applied decision-making situations agents know that they typically have a very small subset of potential information relevant to a decision, including the cumulative motives and positions of others. As a result, they group potential factors into easy to work with categories, reflecting the psychological concepts of compartmentalization and distinction bias. Agents behave in this manner based on the fact that they must interact in a world that reflects the motives of many others. Cumulative counter agent effects serve as a constraint in the decision-making process, not an immovable exogenous constraint, but one against which the agent can only have incremental influence, gradually over time.

Even though agents cannot always explicitly identify quantifiable decision inputs, in terms of C and B, they generally make decisions based on their best possible approximations, through the effort of compartmentalizing based on experience. The principle of cumulative counter agent effects reflects the complex system nature of many interacting agents (Arthur et al., 1997), wherein the aggregate impact of the iterative actions of many agents acting on beliefs that have been incrementally built up over time creates a slow moving system in which the influence of a particular decision-making agent and a particular decision is itself small and incremental. The complex system of interaction is not static and is more than the sum of its parts (Orrell and McSharry, 2009). It will ebb and flow and move as the iterative impacts of individuals build in particular directions. In addition, because all agents within the system are acting in self-interest, the principle of cumulative counter agent effects also means that the system will incrementally move against any agent that does not assertively act in their own self-interest or have someone do so on their behalf. These effects can be modelled with complex economic nonlinear dynamics (Rosser, 1999). A very important corollary follows from the principle of Cumulative Counter Agent Effects, known as the principle of *Return on Cumulative Effort*.

***The principle of return on cumulative effort***

The Principle of Return on Cumulative Effort establishes that decision-makers generally realize that more personal total cost is typically required to obtain more personal total benefit, and that higher levels of personal total benefit can be obtained in the long-run, as a result of a sustained investment of well-directed personal total cost. This is what drives an agent to incrementally and gradually push forward with decisions and actions to eventually achieve their longer-term objectives.

Not only do many decision-makers generally make the critical realization that more deliberate and uncertain C is typically required to obtain more B, there is often also an understanding that, in general, overall higher values of personal total benefit are possible in the long-term versus the short-term (Loewenstein et al., 2003). This reflects an understanding of the notion of increased return on cumulative effort, due to practice and experience. As a result, the *Personal Total Benefit/Personal Total Cost Trade-off Curve (Psychological Trade-off Curve)*, will now be formally defined, representing the first fundamental psychological constraint in Causal Economics. It maps C to B. Over the relevant time period 0<t<M and for all –k<i<N, the psychological trade-off constraint function, *c*, is defined such that;

(14) $$B^{C}=c\left(C^{C}\right)$$

The specific functional form is;

(15) $$c_{i}=\left[B_{it}^{C}/\;C_{\;\;it}^{C}\right]\Psi_{\;\;i}^{C}$$

or more specifically;

(16) $$c_{i}:\left[C^{C}\left\{w^{\left(-\right)}\left(q_{Uit}^{C}\right),v^{\left(-\right)}\left(Q_{Uit}^{C}\right),v^{\left(-\right)}\left(Q_{Ait}^{C}\right)\right\},\Psi_{i}^{C}]\to [B^{C}\{w^{\left(+\right)}\left(p_{Uit}^{C}\right),v^{\left(+\right)}\left(P_{Ait}^{C}\right),v^{\left(+\right)}\left(P_{Uit}^{C}\right)\}\right]$$

or equally;

(17) $$c_{i}=\left\{\;\left[w^{\left(+\right)}\left(p_{\;\;Uit}^{C}\right)v^{\left(+\right)}\left(P_{\;\;Uit}^{C}\right)+v^{\left(+\right)}\left(P_{\;\;Ait}^{C}\right)\right]/\left[w^{\left(-\right)}\left(q_{\;\;Uit}^{C}\right)v^{\left(-\right)}\left(Q_{\;\;Uit}^{C}\right)+v^{\left(-\right)}\left(Q_{\;\;Ait}^{C}\right)\right]\right\}\Psi_{i}^{C}$$

The function *c,* as defined in Eq. (14), is a positive, continuous, strictly increasing function that conveys the allowable trade-off of C in pursuit of B over the relevant time horizon. In general, to achieve higher values of V(*f*) in the longer term, agents recognize that in the short-term they will face lower values of V(*f*) as constrained by lower values of c(*f*). Agents also impose a C threshold constraint at their psychological breaking point (Kimble, 1996). The second fundamental psychological constraint in Causal Economics is the Personal Total Cost Threshold;

Local Personal Total Cost Threshold (L) is defined such that;

(18) $$L=Max\left[C^{C}\left\{w^{\left(-\right)}\left(q_{Uit}^{C}\right),v^{\left(-\right)}\left(Q_{Uit}^{C}\right),v^{\left(-\right)}\left(Q_{Ait}^{C}\right)\right\},\Psi_{i}^{C}\right]$$

Only decisions with expected (B,C) pairs above the $B^{C}/C^{C}$ curve will be considered, such that a selected maximum personal total cost threshold, L, as defined in (18), is not exceeded.

**Fig. 1:**
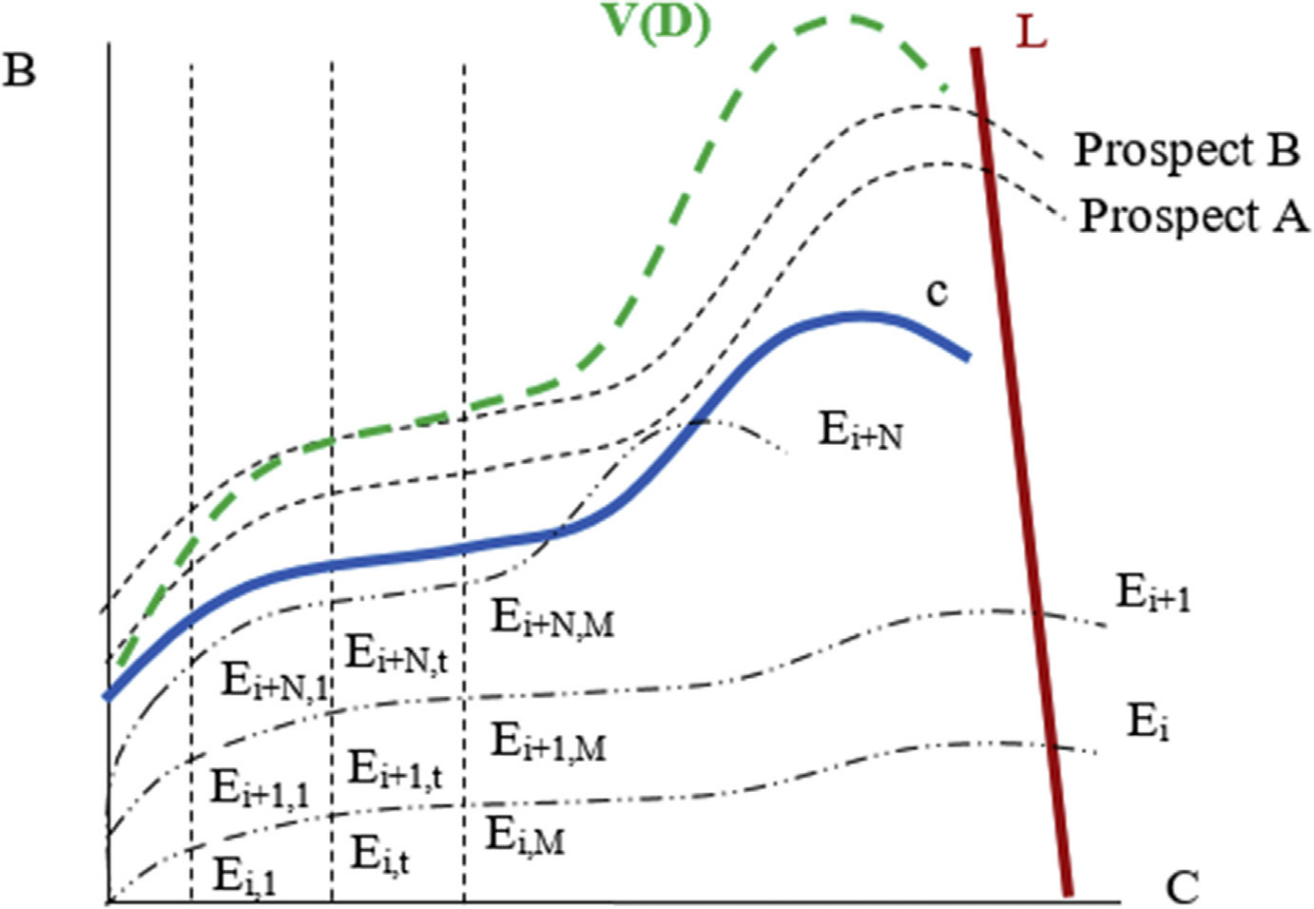
The ‘Leaning X’ shows the trade-off between B and C over the relevant time horizon t[0,M] and captures the relationship of *B*{*w*^(+)^ (*p*_*Uit*_), *v*^(+)^ (*P*_*Ait*_), *v*^(+)^ (*P*_*Uit*_)} and *C*{*w*^(−)^ (*q*_*Uit*_), *v*^(−)^ (*Q*_*Uit*_), *v*^(−)^ (*Q*_*Ait*_)} over all values of *i* and *t*.

Fig. 1 is a core figure in Causal Economics; frequent reference will be made to this *Leaning X* which shows how $B\left\{w^{\left(+\right)}\left(p_{Uit}\right),v^{\left(+\right)}\left(P_{Ait}\right),v^{\left(+\right)}\left(P_{Uit}\right)\right\}$ relates to $C\left\{w^{\left(-\right)}\left(q_{Uit}\right),v^{\left(-\right)}\left(Q_{Uit}\right),v^{\left(-\right)}\left(Q_{Ait}\right)\right\}$ over all values of *i* and *t.*

What is of relevance to the decision-maker is the discounted present value of all future periods (Loewenstein et al., 2003), from M back to the decision-making period, which means that the functions *v*, *w* and *Ψ* each contain a time discounting element. In the Leaning X depiction, the value function is plotted to illustrate its components of C and B at each level of V(*f*) rather than the traditional view of V(*f*) at each outcome value, as utilized in CPT (Tuthill and Frechette, 2002). Whereas the trade-off curve, *c*, represents a range of normal conditions, the L curve represents conditions where an agent is setting their personal break point (Kimble, 1996). This is a psychologically determined value, and agents may find themselves resetting their own break point. The L line has slightly negative slope to capture diminishing sensitivity that exists even when an agent sets an internal maximum threshold. An agent's L will often differ in the long run and short run and over various conditions (including endowment levels).

Travelling North West along the L curve results in higher levels of B relative to C, meaning that as an agent has more B, they are less open to taking on more maximum C. Travelling South East along the L curve results in higher levels of C relative to B, meaning that as an agent has less B they are more open to take on more C in order to obtain even small amounts of additional B, accepting a higher L. The slope of *c* is determined significantly by the impact of cumulative counter agent effects and return on cumulative effort. Agents generally have to decide how much C (effort, spend, foregone opportunity etc.) a particular decision and associated course of action requires, both certain (deliberate) and uncertain, with the expectation that some potential B will result. This is not to say that agents can exactly determine C, since external influences will impact their estimation. External impacts, especially over extended periods of time, can introduce a margin of risk to C.

Because agents directly control a significant portion of their effort and expenditure, the amount of C one bears is usually more certain (and more deliberately incurred) than B, the latter generally being impacted by the efforts/expenditures of other agents, which are less certain to the decision-maker and reveal themselves through Cumulative Counter Agent Effects. Whenever new information or other factors impact the parameters of the decision model the decision-making process is repeated (Camerer et al., 2011).

**Fig. 2:**
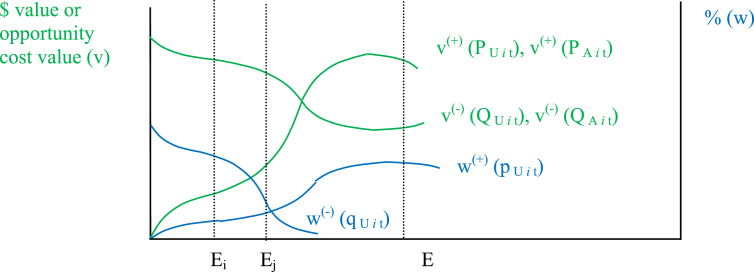
Typical slopes of w^(+)^ (p_U*it*_), w^(−)^ (q_U*it*_), v^(+)^ (P_U*it*_), v^(−)^ (Q_U*it*_), v^(+)^ (P_A*it*_), v^(−)^ (Q_A*it*_) over E, where E_i_ < E_j_.

For modeling purposes, Fig. 2 shows that $v^{\left(+\right)}\left(P_{Ait}\right),v^{\left(+\right)}\left(P_{Uit}\right)$ and $w^{\left(+\right)}\left(p_{Uit}\right)\;$ are assumed to be continuous and strictly increasing over time throughout most of their range, whereas $v^{\left(-\right)}\left(Q_{Uit}\right),v^{\left(-\right)}\left(Q_{Ait}\right)$ and $w^{\left(-\right)}\left(q_{Uit}\right)$ are assumed to be continuous and strictly decreasing over time throughout most of their range. Endowments, reflecting wealth levels, impact the nature of the psychological trade off relationship. As wealth levels increase, a higher level of B naturally flows to the agent in most decision scenarios, resulting in a higher marginal rate of trade-off of B required for each additional unit of C. This reflects the principle of diminishing sensitivity (Erev et al., 2008) as it applies to B.

### 2.5 Incentives

Agents have an incentive to act whenever, X, the Causal Coefficient, is greater than 1;

(19) $$X\;=B^{\prime }/C^{\prime }\;>1\;$$

The Causal Coefficient (19) represents the incremental rate of change in B relative to the incremental rate of change of the C that causes it within a particular alternative. When X≥1 for all agents, Causal Coupling is achieved.

### 2.6 Disincentives

Agents have a disincentive to act whenever, X, the Causal Coefficient, is less than 1;

(20) $$X\;=B^{\prime }/C^{\prime }\;<\;1\;$$

### 2.7 Characterizing attitudes toward risk & personal total cost

Many decision-making models that address uncertainty rely solely on the assumption of risk aversion. However, empirical research has demonstrated that this formulation does not account for the observed asymmetry between attitudes toward gains versus losses, an asymmetry that exceeds what can be adequately explained via income effects or an assumption of decreasing risk aversion (Kahneman and Tversky, 1979).

Decisions that reflect ‘risk-seeking’ behaviour are observed in two common situations. Decision-making agents often exhibit preference for a relatively small possibility of obtaining a relatively large positive outcome value over the expected value of the prospect (Kahneman and Tversky, 1979). Risk-seeking behaviour is also common in situations where decision-making agents are able to choose between a completely certain loss and a substantial probability of a larger loss (Kahneman and Tversky, 1979). This latter ‘risk seeking’ behaviour reflects an agent's desire to try to avoid the certain loss if at all possible.

Many economic models equate risk to variability (Engle, 2004). Variability is a mathematical measurement that includes risk through potential C, and potential rewards from taking on that risk in the form of B. Psychology predominantly aligns risk only to loss (Duxbury and Summers, 2004). The latter approach is taken in Causal Economics, where risk is equated to the potential of uncertain C to the agent. Reward from risk, or ‘upside’, is not risk, even though it is a component of variability. Some key definitions result;

(21) $$\begin{array}{l} \begin{array}{l} Variability=Uncertainty\\ Variability=Risk+Reward\;from\;Risk\\ Risk<Variability \end{array}\\ Risk=\;C_{R}\left\{w^{\left(-\right)}\left(q_{Uit}\right),v^{\left(-\right)}\left(Q_{Uit}\right)\right\} \end{array}$$

Associated with the uncertain components of C and B are distributions of potential values. Agents will seek the reward from risk, prepared to bear the causally-linked risk as a necessary cost. Cumulative Prospect Theory captures the existence of both risk aversion and risk seeking (variability seeking) behavior by providing that risk seeking/risk aversion dynamics are jointly determined by the value function and the probability weighting function (Tversky and Kahneman, 1992). Causal Economics broadens the scope of consideration even further in order to model both attitudes toward risk as well as attitudes toward C—the latter including risk and certain C.

As a result, in Causal Economics, overall C aversion is determined by the *C Tolerance Ratio*, 1/*c'*. This ratio captures how much additional C one will take on in order to obtain an expected additional unit of B;

(22) $$Personal\;Total\;Cost\;Tolerance\;Ratio=1/c^{\prime }$$

Variability seeking/risk aversion are determined by the *Risk Tolerance Ratio*, 1/r', as defined in Eq. (22), which isolates the uncertain component of C. To isolate an agent's attitude toward risk from overall variability, the certain components are removed, transforming the C tolerance ratio by isolating the uncertain elements of C and B. Risk perspectives have a time profile, so the entire decision time horizon must be considered.

Over all values of *i*, where –k<i<N and *t*, where 0<t<M, we define;

(23) $$r:C_{R}\left\{w^{\left(-\right)}\left(q_{Uit}\right),v^{\left(-\right)}\left(Q_{Uit}\right)\right\}\;\to \;B_{R}\left\{w^{\left(+\right)}\left(p_{Uit}\right),v^{\left(+\right)}\left(P_{Uit}\right)\right\}\;\&\;C_{R}\left\{w^{\left(-\right)}\left(q_{Uit}\right),v^{\left(-\right)}\left(Q_{Uit}\right)\right\}<\;L_{R}$$

This provides a definition of the agent's risk tolerance ratio;

(24) $$Risk\;Tolerance\;Ratio=1/r^{\prime }\;$$

The value function, *v*, the probability weighting function, *w*, and the overall rank dependent outcome weighting function, *Ψ*, each contribute as components into 1/c' *and* 1/r' (24). Breaking risk and C acceptance attitudes into these components allows rich modeling of the relative influence and interaction of risk preference and sensitivity to C and B levels. An agent's attitude toward risk (Tversky and Kahneman, 1992) reflects the interplay of judgments about gains versus losses, outcome magnitudes, probabilities, distance from reference point (such as the status quo), as well as current and expected endowments.

As discussed previously, two key traits of the Causal Economics value function are taken from CPT in order to underpin inherent natural overall risk aversion in decision-making agents. Firstly, over most of its range the losses portion of the curve is convex, implying that people are motivated more by losses than by gains and as a result will devote more energy to avoiding loss than to achieving gain (Kahneman et al., 1991). Secondly, the value function is steeper for losses than it is for gains (Tversky and Kahneman, 1992). These factors are themselves impacted by the time horizon under consideration. Over longer time horizons of consideration, the value function and the psychological trade-off function each transition from generally concave to convex and back to concave, as sensitivity and risk aversion interact with cumulative counter agent effects.

In exchange for taking on risk, agents expect a return, defined as;

(25) $$Expected\;Payoff\;from\;Risk=B_{R}\left\{w^{\left(+\right)}\left(p_{Uit}\right),v^{\left(+\right)}\left(P_{Uit}\right)\right\}$$

As defined previously, risk is the expected impact of the uncertain potential downside, the uncertain component of personal total cost. Speculation is defined when the agent deliberately takes on risk in pursuit of payoff from that risk, expecting both reward and risk possibilities in excess of the certain part of C and B.

#### 2.7.1 Speculation

Speculation is defined when an agent deliberately takes on risk in pursuit of payoff from that risk, expecting both reward and risk possibilities in excess of the certain part of personal total cost and personal total benefit, with reward from risk exceeding risk taken;

(26) $$B_{R}\left\{w^{\left(+\right)}\left(p_{Uit}\right),v^{\left(+\right)}\left(P_{Uit}\right)\right\}>C_{R}\left\{w^{\left(-\right)}\left(q_{Uit}\right),v^{\left(-\right)}\left(Q_{Uit}\right)\right\}>0$$

Since agents cannot know with certainty the B versus C trade-off functions of other agents, they cannot know the future decisions or actions of individuals with whom they may potentially interact. There will in effect be some positive level of uncertainty, which means that $w^{\left(-\right)}\left(q_{Uit}\right)>0$ and $w^{\left(+\right)}\left(p_{Uit}\right)>0$. Every agent faces the dilemma of not knowing whether the status quo or a particular different action into the uncertain will yield greater B net of C. As a result, each agent will *test*, which means that in the face of outcomes with uncertainty they will ‘put their toe in the water’ to a degree of comfort in line with their risk tolerance and overall C tolerance. They will take on some small level of known $C\left\{v^{\left(-\right)}\left(Q_{Ait}\right)\right\}$ and known $B\left\{v^{\left(+\right)}\left(P_{Ait}\right)\right\}$ and risk $\left\{w^{\left(-\right)}\left(q_{Uit}\right),v^{\left(-\right)}\left(Q_{Uit}\right)\right\}$ to see whether new information gained from the resulting event of that decision/action produces new expectations of higher B relative to C in the future. This is captured in Eq. (26). Agents always bear uncertainty, and so no matter how risk averse, they will have some level of acceptable uncertain C less than L that they are willing to take on in order to test. As an agent becomes more risk averse, L approaches zero as risk aversion approaches ∞.

#### 2.7.2 Testing behaviour

Whenever risk is perceived to exist, $w^{\left(-\right)}\left(q_{Uit}\right)>0$ testing will occur as agents will take on a small amount of additional risk $\left\{v^{\left(-\right)}\left(Q_{Uit}\right)<{Є}_{Q},w^{\left(-\right)}\left(q_{Uit}\right)<{Є}_{q}\right\}$ in addition to certain personal cost $v^{\left(-\right)}\left(Q_{Ait}\right)$ and certain personal benefit $v^{\left(+\right)}\left(P_{Ait}\right)$ in order to see whether they can obtain additional uncertain personal total benefit (reward from risk) $\left\{v^{\left(+\right)}\left({P_{Ui}}_{t}\right),w^{\left(+\right)}\left({p_{U}}_{it}\right)\right\}$, as long as c′>1 and such that $C\left\{w^{\left(-\right)}\left(q_{Uit}\right),v^{\left(-\right)}\left(Q_{Uit}\right),v^{\left(-\right)}\left(Q_{Ait}\right)\right\}<\;L$. Testing behaviour is the underlying core of speculation.

### 2.8 Psychological foundations of the trade-off constraint

Many psychological principles that have been experimentally established serve to provide scientific evidence of the existence of the psychological trade-off curve. *Cognitive Dissonance* is a vital reason that the psychological trade-off curve exists. Significant efforts have been made to incorporate this principle into formal economic models (Akerlof and Dickens, 1982). The concept is characterized by agents maintaining a particular belief that has been held for a long time, even when it is at odds with new evidence (Festinger, 1957). This behaviour reflects the fact that agents trust what they have learned over their lifetime of experience, as opposed to a single observation, in determining expected personal total C and B. Experiences are anchored (Mussweiler and Strack, 2001) through repetition and as they are built up over time they become more certain to the decision-maker. Each agent's psychological trade-off curve incorporates the expected impact of cumulative counter agent effects, giving short-to-intermediate-term permanence to the curve.

Closely related to the concept of cognitive dissonance is that of *Status Quo Bias* (Samuelson and Zeckhauser, 1988). Experiments show (Kahneman et al., 1991) that agents will typically not change an established behaviour unless the incentive to change is significant. Any outcome other than the status quo is most likely to represent heightened uncertainty and increased (and largely deliberate) exposure to C, at least in the short-term. As discussed previously, this front-loading of C with eventual resulting B is captured in the return on cumulative effort corollary of the cumulative counter agent effects principle. When an agent perceives that doing nothing is likely to lead to drastically negative outcomes, conditions for change are in place.

The psychological concept of *Compartmentalizing* (Potts et al., 1989) not only serves to ground the axiom of Causally Coupled outcomes, but combined with the psychological principle of *Distinction Bias* (Hsee and Leclerc, 1998), also helps underpin transitive, rank dependent preferences. Distinction bias is the tendency to view two options as more dissimilar when they are evaluated simultaneously rather than separately, which also allows agents to make the ordinal category rankings that underpin their preference ranking. Distinction bias drives agents to make distinctions, because alternatives are being evaluated side-by-side (Hsee and Leclerc, 1998). This supports a well-defined preference ordering, defined across overall weighted outcome values (events) relative to other possible events. The combination of compartmentalizing and distinction bias also provides strong psychological grounding for use of the sign-comonotonic trade-off consistency criterion which essentially replaces the Expected Utility independence axiom of Cumulative Prospect Theory. A thorough axiomatization of sign-comonotonic trade-off consistency has been provided by Wakker and Tversky (1993).

Cumulative Prospect Theory posits that decisions are weighed as gains or losses relative to the status quo scenario (Fennema and Wakker, 1997). Causal Economics does not absolutely require this condition, as the selection of a benchmarking point is a subjective decision personal to each agent. Many agents will relate outcomes to the current status quo, whereas many others will relate to zero and still other highly motivated and proactive agents will reference against some target scenario they see resulting in the future as the result of their continued action. A number of related psychological principles give further weight to the resilience of the psychological trade-off curve as a realistic representation of decision-making optimization. The *Framing* concepts of *Selective Perception* and *Confirmation Bias* (Tversky and Kahneman, 1981) in particular have the effect of anchoring and perpetuating the trade-off curve relationship as an agent makes more and more decisions over time.

Selective perception (Massad et al., 1979) is well documented in psychology and results in a tendency of the expectations of agents to actually affect their perception of potential outcomes. In essence, this implies that the trade-off relationship agents possess is applied to new situations, making it in many ways self-fulfilling. This behavioural driver is further strengthened by the existence of confirmation bias, as agents actually search for and interpret information in a manner that confirms their existing preconceptions. Agents in effect frame their perspective, reflected through their perceptions of C and B, which further perpetuates through projection to future decision scenarios.

Longer time horizons are a major driver of increased levels of uncertainty, because they can introduce many new impacts, potentially even requiring a reset of an agent's preference rankings. *Hyperbolic Discounting* is a psychological principle that succinctly captures the impact of various time horizons, due to changing risk profiles and preference. The principle notes that an individual will have a stronger preference for more immediate payoffs relative to later payoffs (Green et al., 1994). In addition, this preference itself accelerates as all payoffs are closer to the present. The psychological concept of *Recency* must also be considered (Miller and Campbell, 1959). It confers that agents place the greatest emphasis on more recent information and either ignore or forget to consider distant information. This psychological principle has a major impact on decisions within Causal Economics, because it means that agents may apply a particular local maximum personal total cost threshold (L) in the short-term and then revisit their decision with a different maximum L in the longer term.

To illustrate the importance of the internal psychological C versus B constraint concept, consider a practical, applied example. By ridding oneself of a fear of heights, an agent could presumably instantly increase utility, by enjoying height-related activities without discomfort. Given a very minute actual risk of death in most situations, an agent's fear of heights is in general taking potential B ‘off the table’. This is where a psychological understanding of behaviour better grounds the economic model of utility maximization and decision-making. Agents can change their associations over time, but a lifetime of experience and conditioning makes this beyond the scope of a single economic decision at hand.

The C versus B trade-off is further supported by observations of neurobiology. The Somatic-Marker hypothesis (Damasio et al., 1991) asserts that within the context of uncertainty, when considering potential future outcomes, agents simplify their decision-making process with the aid of emotions (in the form of bodily states). These bodily states distinguish alternative decisions/actions as being advantageous (i.e. delivering overall B) or disadvantageous (i.e. delivering overall C). This neurobiological survival mechanism motivates agents to be cautious and incrementally learn, advancing at a managed level of risk. The Somatic-Marker hypothesis provides further pluralistic underpinning for the psychological trade-off constraint.

### 2.9 Optimization

Agents make a decision, λ, by selecting the alternative mindset, course of action or course of inaction that yields the associated highest subjectively valued prospect over the expected relevant time horizon;

(27) λ=MaxV(f)

In making a decision an agent chooses a prospect over all values of *i* and *t*, to bear a particular certain (deliberate) impact $v^{\left(-\right)}\left(Q_{Ait}\right),v^{\left(+\right)}\left({P_{A}}_{it}\right)$ and a causally associated uncertain impact captured via the functions $w^{\left(+\right)}\left(p_{Uit}\right),v^{\left(+\right)}\left(P_{Uit}\right),w^{\left(-\right)}\left(q_{Uit}\right)\;and\;v^{\left(-\right)}\left(Q_{Uit}\right)$. The result is a conditional judgement, λ, defined as;

(28) $$\lambda =\left\{w^{\left(+\right)}\left(p_{Uit}\right),v^{\left(+\right)}\left(P_{Uit}\right),w^{\left(-\right)}\left(q_{Uit}\right),v^{\left(-\right)}\left(Q_{Uit}\right)\;|v^{\left(-\right)}\left(Q_{Ait}\right),v^{\left(+\right)}\left(P_{Ait}\right)\right\}$$

The optimization process in Causal Economics is therefore a maximization of;

(29) $$\begin{array}{l} V\left(f\right)=\;V^{+}\left\{w^{+\left(+\right)}\left(p_{Uit}\right),v^{+\left(+\right)}\left(P_{Uit}\right),v^{+\left(+\right)}\left(P_{Ait}\right),w^{+\left(-\right)}\left(q_{Uit}\right),v^{+\left(-\right)}\left(Q_{Uit}\right),v^{+\left(-\right)}\left(Q_{Ait}\right),\Psi_{i}^{+}\right\}+\\ V^{-}\left\{w^{-\left(+\right)}\left(p_{Uit}\right),v^{-\left(+\right)}\left(P_{Uit}\right),v^{-\left(+\right)}\left(P_{Ait}\right),w^{-\left(-\right)}\left(q_{Uit}\right),v^{-\left(-\right)}\left(Q_{Uit}\right),v^{-\left(-\right)}\left(Q_{Ait}\right),\Psi_{i}^{-}\right\} \end{array}$$

Subject to;

(30) $$c:\left[C^{C}w^{\left(-\right)}\left(q_{Uit}^{C}\right),v^{\left(-\right)}\left(Q_{Uit}^{C}\right),v^{\left(-\right)}\left(Q_{Ait}^{C}\right)\},\Psi_{i}^{C}]\to [B^{C}\{w^{\left(+\right)}\left(p_{Uit}^{C}\right),v^{\left(+\right)}\left(P_{Ait}^{C}\right),v^{\left(+\right)}\left(P_{Uit}^{C}\right)\}\right]\;and$$

(31) $$Max\left[C^{C}\left\{w^{\left(-\right)}\left(q_{Uit}^{C}\right),v^{\left(-\right)}\left(Q_{Uit}^{C}\right),v^{\left(-\right)}\left(Q_{Ait}^{C}\right)\right\},\Psi_{i}^{C}\right]\;=\;L$$

Only prospects (29) that are at all points on or above the trade-off curve, *c,* as defined by (30), will be considered, such that the maximum personal total cost threshold, L, as defined by (31), is not exceeded. It is important to always consider the status quo, or inaction, as an alternative to be weighed against other potential decision alternatives (Kahneman et al., 1991). The status quo will have its own associated C and B values for the agent.

Of additional importance is the reality that decision-making occurs a priori to observed outcomes. Potential B and C are therefore anticipated as best as possible. Decisions are impacted by previous decisions and observed outcomes. Decisions are typically sequential (Arrow and Fisher, 1974), requiring associated follow-through actions and subsequent reaffirming decisions to deliver meaningful results. For example, the decision to lose weight is actually a high level macro decision, followed by a sequence of related decisions, as an agent must decide each and every day what to eat and how much to exercise. Many agents will also routinely underestimate C, based on overconfidence (Blanton et al., 2001), and find themselves re-evaluating once action has been taken and results observed. This captures the notion of ‘second guessing’, and contributes to iterative decision-making.

**Fig. 3:**
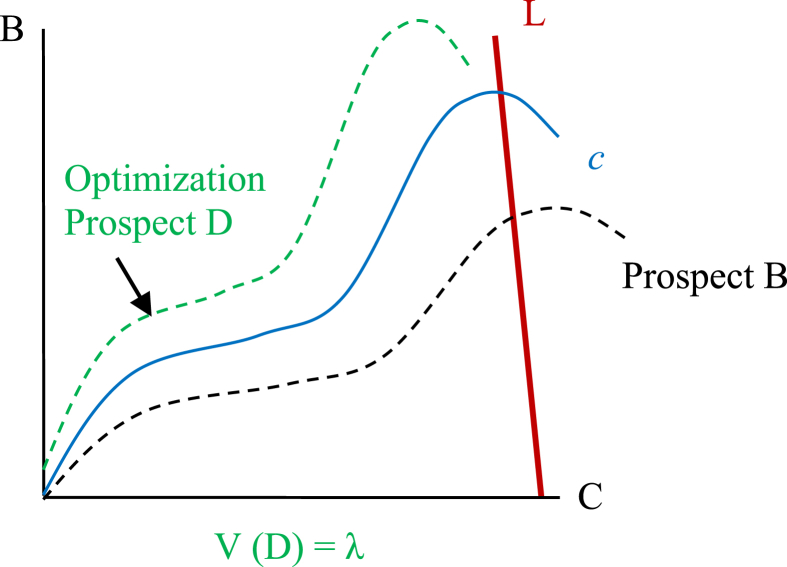
Graphical representation of target decision: The ‘Leaning X’.

Fig. 3 (the ‘Leaning X’) demonstrates the optimization process of an agent in making a decision and in selecting a course of action, but it does not represent an equilibrium. Equilibrium can never exist in real world situations given the existence of the testing principle and levels of uncertainty greater than zero.

**Fig. 4:**
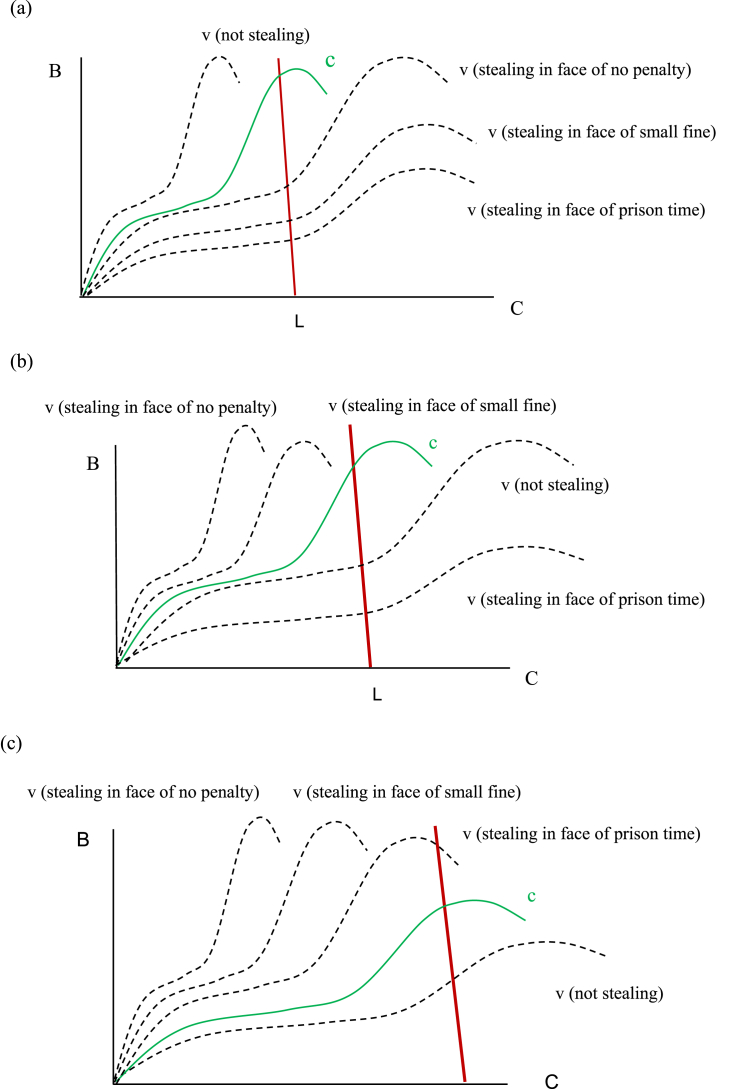
Reactions to a penalty in the face of potential criminal ‘opportunity’. (a) Perspective of a law abider (b) perspective of a petty criminal (c) perspective of a hardened criminal.

Fig. 4 compares how individuals with differing perspectives will respond to scenarios that reflect various combinations of B and C.

As we would expect, this example demonstrates that the C of serious crimes must be large enough relative to B to deter the hardened criminal, not the law abiding citizen. These diagrams are shown a priori to a decision/action to steal or not to steal, and as a result, the schematics could differ significantly under various scenarios. For example, an agent's perspective will most often differ if they have recently been caught stealing or if they have experienced a long string of stealing successes without punishment.

## 3 Results & discussion

### 3.1 Social coordination & social coordination failure

Contemporary economic theory provides thorough analysis of the extent to which markets achieve efficient coordination and the extent to which they sometimes fail (Bator, 1958), bringing about market failure and subsequent problems such as moral hazard, free riding and externalities in general. Causal Economics provides for convenient expansion of the well-entrenched concepts of a market and market failure to a broader context that models social coordination and social coordination failure.

#### 3.1.1 Social coordination failure

Social coordination failure is a generalization of market failure. It occurs when C and B are causally decoupled (X<1) across individuals involved or impacted by any economic/social activity.

Causal Economics delivers a bold conclusion—that essentially every economic social coordination problem (defined as delivering non-Pareto Optimal outcomes) within societies can be traced back to a causal decoupling of B and C (20) across populations of involved or impacted agents, and that effective solutions can be uncovered by ensuring that causal coupling is achieved (19). Social coordination problems generally result when some agents are able to pursue their personal enrichment without accountability to society, where some agents gain B through means other than a voluntary interaction/exchange (by avoiding some or all of the associated C). When causal decoupling occurs, inefficient, non-Pareto Optimal societal allocations result.

Causal Coupling (19) does not mean that C and B are equated at the transaction or aggregate level, as all agents have different interpretations that determine their values of B and C and since B in excess of C is required in order to provide an incentive for agents to act. Causal Coupling of B and C means that agents and intermediaries are not able to obtain B at the involuntary disproportionate C of others, taking into account typical preferences, usage and risk. Where involuntary allocations occur through taxation to fund democratically determined public goods/services and public risk sharing programs etc., allocations of certain and uncertain B and C should be prorated across individuals. This means every agent that obtains B bears the associated (causally coupled) C, either through voluntary action and exchange or through proportionate allocation of total costs and total benefits (certain and uncertain) in the case of public services, including population-wide risk sharing/insurance programmes.

Market failure occurs when the voluntary actions of agents fail to take into account broader indirect impacts, beyond the current transaction. This is essentially a decoupling of C and B over the intermediate to long-term. Causal Economics abandons the concept that market failure is due to exogenous externalities in favour of the concept that it is due to endogenously generated causal decoupling between C and B across the population.

### 3.2 Four primary causal coupling mechanisms

Pareto optimal solutions combine the four approaches shown below:

1. **Voluntary Trade/Agreements Adjusted for Externalities:** This should represent the vast majority of interactions in a free society. The voluntary nature of the truly free market/agreements significantly aligns B and C across individuals, barring externalities. The impact of the latter should be allocated through one or more of the other mechanisms.
2. **Equal Share Public Services**: Infrastructure that similarly benefits every citizen in line with democratic decisions should be funded via a flat tax allocation across all individuals. Military expenditure serves as an example. Where citizens can't financially afford to cover their allocation of the cost burden, they should be able to provide their labour in lieu via valuefare (workfare at market wages with option to select number of hours worked).
3. **User Specific Public Services:** Where individuals utilize more than their equal share of society-wide services or a specific service not used by all individuals, a user fee should be collected. Toll roads serve as a simple example in this area.
4. **Public Risk Sharing:** Where individuals in society face low frequency, high magnitude risks from societal impacts, C premiums should be allocated across all individuals via insurance. Individual premiums should be risk-adjusted to reflect individual risk profiles above or below average. Mandatory third party liability auto insurance serves as an example in many jurisdictions.

Approaches toward the top of this list most tightly couple costs and benefits across individuals and society. Those toward the end rely on broader allocation, which means programs must be designed and deployed to preserve maximum causal coupling. The four solution approaches together ensure that effective causal coupling is built in at the core—they are essentially the practical design elements. Principles such as self-interest, adverse selection and moral hazard ensure that any large scale social programs face their share of challenges. Program policing is required to keep them effective and viable, both organizationally and in meeting the needs of their stakeholders.

Voluntary activity is by far the best core coordination approach within free and democratic societies. However, voluntary activity can create significant externalities upon other individuals and the broader environment. Physical and system infrastructure is also often required to support free commerce—legal systems and utilities to name a few. Direct levies are required to cover these costs and for use of government services and risk programs. Ensuring that citizens who benefit from large scale societal programs pay their share of associated costs is a large challenge.

Voluntary activity with policing goes a long way to keeping behaviours in line. Insurance is the final key tier of programs that effectively achieve causal coupling in practice. It's critical because there will always be cases where some users obtain more B than the C they contributed through non-voluntary interactions. This is especially true for risk sharing programs, such as core health care. For example, there will always be individuals that smoked, got lung cancer and didn't save anything for treatment, even though they should pay a larger share of health costs. Risk sharing insurance is fundamental in many areas, such as mandatory automobile insurance and in core healthcare areas like access to emergency services.

It is not cost effective to have 100% policing on all activities, and citizens of a free democracy would typically not tolerate this level of intrusion and infrastructure cost. Effective systems must give citizens the freedom to comply with the knowledge that policing and actual consequential penalties apply to breaches. Western legal systems for the most part serve as a good example. Policing works, but there are significant issues with how effectively the system attaches penalties to breaches and costs to spurious litigation.

### 3.3 Applications

Causal Economics and its core concept of Causal Coupling reinforce the Pareto Optimality of scenarios that prioritize individual freedom of choice with responsibility to society. This introductory paper only scratches the surface with potential applications to stimulate new ideas. In practice, Causal Economics broadly makes its strongest case against the economic policies of the left side of the political spectrum. This is because Causal Coupling is seriously undermined where there are large governments made up of career politicians that cater to lobbies and sustain themselves on persistent redistributive tax collection. As an example, Causal Economics does not support income, consumption or wealth taxes. In their place it supports specific and transparent flat tax allocations and user fees to maximize causal coupling of B and C.

Income, consumption and wealth taxes break causal coupling because they fill the coffers of government automatically (B without explicit C). The government receives funding and compensation that is not tied to results. It becomes reliant on money just coming in, and doesn't have to raise funds for each spending program. Even worse, these taxes break causal coupling because they introduce a distortionary cost on income-producing transactions in the economy, lowering output. User fees causally couple the cost of the fee to the benefit the user obtains as directly as possible. Flat taxes spread the cost of a program across all members of society that benefit. As a result, flat taxes and user fees maintain casual coupling as much as possible for services that apply to the vast majority of citizens (determined by democratic vote) and specific users of services respectively.

The lens of Causal Coupling also provides clear and refreshing insight into the proper foundations of important social programs provided by government. It shows us that Causal Coupling is only maintained when social programs provide direct critical support without obstacle, and then tie opportunities for greater benefits to additional contributions to society. This is why workfare programs can work and welfare programs without improvement incentives built in will only create long-term poverty and dependence. This reflects basic human incentives that apply to all people. Unfortunately the politics around such an issue often cloud the reality necessary to improve conditions. Causal Economics opens our eyes to innovative new ideas for social assistance, such as allowing citizens to work as many hours as they want for an hourly workfare wage (Valuefare). They can be given some direct tasks that would normally be done by unskilled career government employees (another saving) and/or be directly compensated for re-training. Overall tax payers will pay less, low-income citizens willing to contribute get ahead and those that are not interested in contributing will self-select to stay unproductive and lower-income without higher cost to society. This example shows the power of restructuring a system to align to Causally Coupled incentives.

The areas of risk sharing and insurance also benefit from the approach of Causal Economics. Like goods and services, free markets for insurance will take care of the vast majority of needs. However, there is still a role for societal risk sharing, especially where individuals face limited access to private insurance. Where certain identifiable citizens face higher than average risk, their associated costs can be pooled to reduce potential spikes for all members of the group. Some risk situations, like car insurance, face adverse selection. This means that the people who don't need insurance as much still buy it and those that should buy it, due to high risk profiles, decide not to buy it. Mandatory insurance can be required in such situations because the costs of an accident to others in society can be prohibitive. When a risk scenario impacts virtually every citizen, the cost of pooling the risk and the benefit of spreading out that risk are allocated evenly across citizens and causal coupling is achieved.

Even though Causal Coupling asserts that freedom and free markets must be at the core of all effective policies and institutions, the theory still highlights necessary accountabilities for citizens that put some limits on the arguments of the right side of the political spectrum. Specifically, free market interactions often don't address important externalities that affect citizens beyond a transaction. In such cases, like damage to the environment, there will need to be corrective measures such as an environmental cleanup user fee.

An unfettered free market profit motive also isn't feasible for large societal infrastructure projects, because it would also severely breach Causal Coupling. These things do require government, but by no means large, self-rewarding, automatically-funded, unaccountable government. Government can be small, transparent and accountable (through compensation tied to performance, not just elections). The same issue of large and powerful self-rewarding government applies to capitalist business. At massive scale, businesses can often talk a great free market story at the same time as they coerce government for preferential treatment and rewrite rules to squash competitors.

In practice, intermediaries across the spectrum—government, big business, big labour and big interest groups—all find ways to self-sustain and prosper in a system that the everyday citizen funds. Large intermediaries and concentrated power at the top are truly what citizens must be vigilant toward. The fundamental takeaway of Causal Economics in the real world is that effective solutions center on freedom for the individual first and foremost, with appropriate accountability to society. The theory predicts that societies with pervasive de-coupling are inherently unstable, which provides for an interesting line of research into potential stability/instability metrics.

## 4 Conclusion

Casual Economics provides a new powerful and pluralistic theoretical model and applied framework to address classic and contemporary economic and social issues, continuing with the breakthrough insights of behavioral economics. The Leaning X diagram (Fig. 1) allows modelling of decisions in a simple way that brings together the full richness of psychological trade-offs with the analytical rigour of economics. C and B causations can be mapped out for each alternative and the Causal Coefficient (X), as defined by Eq. (19), can be used to determine whether a particular decision is optimal for all individuals involved or impacted. Government policies or any other situations that leave all agents with X≥1 or X=0 (not impacted) are Pareto optimal and sustainable. Those that result in some agents experiencing X<1 are not optimal and sustainable in the long-term.

Free market trade and other voluntary interaction is always at the core of effective causal coupling. Where externalities are generated, X≥1 can be achieved across all individuals involved through user fees, prorated levies and or insurance programs. In today's heated and divided political times, new approaches are needed. Causal Economics delivers some bold new conclusions at an exciting time in the field of economics. The model provides a much-needed pluralist framework for additional research into the optimality of concepts that prioritize individual freedom of choice and responsibility to society. Tremendous opportunity exists for additional research into this pluralist framework of behavioral economics as well as for pragmatic application to pressing and controversial real world issues. A particularly rich opportunity for exploration exists in measuring intermediary power and pervasive de-coupling through metrics that can signal economic/societal stability/instability.

## Declarations

### Author contribution statement

Andrew Horton: Conceived and designed the analysis; Analyzed and interpreted the data; Contributed analysis tools or data; Wrote the paper.

### Funding statement

This research did not receive any specific grant from funding agencies in the public, commercial, or not-for-profit sectors.

### Competing interest statement

The authors declare no conflict of interest.

### Additional information

No additional information is available for this paper.
